# Role of aldehyde dehydrogenase in hypoxic vasodilator effects of nitrite in rats and humans

**DOI:** 10.1111/bph.13122

**Published:** 2015-04-29

**Authors:** Sayqa Arif, Alessandra Borgognone, Erica Lai-Sze Lin, Aine G O'Sullivan, Vishal Sharma, Nigel E Drury, Ashvini Menon, Peter Nightingale, Jorge Mascaro, Robert S Bonser, John D Horowitz, Martin Feelisch, Michael P Frenneaux, Melanie Madhani

**Affiliations:** 1 Centre for Cardiovascular Sciences, College of Medical and Dental Sciences, University of BirminghamBirmingham, UK; 2 Department of Cardiology, Royal Liverpool University HospitalLiverpool, UK; 3 Wellcome Trust Clinical Research Facility, Queen Elizabeth HospitalEdgbaston, Birmingham, UK; 4 Department of Cardiothoracic Surgery, Queen Elizabeth Hospital, University Hospitals BirminghamEdgbaston, Birmingham, UK; 5 Basil Hetzel Institute, Queen Elizabeth Hospital, University of AdelaideAdelaide, Australia; 6 Clinical and Experimental Sciences, Faculty of Medicine, University of SouthamptonSouthampton, UK; 7 School of Medicine and Dentistry, University of AberdeenAberdeen, UK

## Abstract

**Background and Purpose:**

Hypoxic conditions favour the reduction of nitrite to nitric oxide (NO) to elicit vasodilatation, but the mechanism(s) responsible for bioconversion remains ill defined. In the present study, we assess the role of aldehyde dehydrogenase 2 (ALDH2) in nitrite bioactivation under normoxia and hypoxia in the rat and human vasculature.

**Experimental Approach:**

The role of ALDH2 in vascular responses to nitrite was studied using rat thoracic aorta and gluteal subcutaneous fat resistance vessels from patients with heart failure (HF; 16 patients) *in vitro* and by measurement of changes in forearm blood flow (FBF) during intra-arterial nitrite infusion (21 patients) *in vivo*. Specifically, we investigated the effects of (i) ALDH2 inhibition by cyanamide or propionaldehyde and the (ii) tolerance-independent inactivation of ALDH2 by glyceryl trinitrate (GTN) on the vasodilator activity of nitrite. In each setting, nitrite effects were measured via evaluation of the concentration–response relationship under normoxic and hypoxic conditions in the absence or presence of ALDH2 inhibitors.

**Key Results:**

Both in rat aorta and human resistance vessels, dilatation to nitrite was diminished following ALDH2 inhibition, in particular under hypoxia. In humans there was a non-significant trend towards attenuation of nitrite-mediated increases in FBF.

**Conclusions and Implications:**

In human and rat vascular tissue *in vitro*, hypoxic nitrite-mediated vasodilatation involves ALDH2. In patients with HF *in vivo*, the role of this enzyme in nitrite bioactivation is at the most, modest, suggesting the involvement of other more important mechanisms.

## Tables of Links

**Table:** 

TARGETS	
**GPCRs**^a^	**Enzymes**^b^
AT2 receptor	Angiotensin converting enzyme 1 (ACE1)
	Aldehyde dehydrogenase 2 (ALDH2)
	Endothelial NOS (eNOS)

**Table:** 

LIGANDS	
ACh	Nitric oxide (NO)
Aspirin	PGF2α
Cyanamide	Phenylephrine (PE)
Glyceryltrinitrate (GTN)	Spironolactone
Lidocaine	

These Tables list key protein targets and ligands in this article which are hyperlinked to corresponding entries in http://www.guidetopharmacology.org, the common portal for data from the IUPHAR/BPS Guide to PHARMACOLOGY (Pawson *et al*., 2014) and are permanently archived in the Concise Guide to PHARMACOLOGY 2013/14 (*^a,b^*Alexander *et al*., 2013a,b).

## Introduction

Nitrite can be chemically reduced *in vivo* to nitric oxide (NO) to elicit vasodilatation (Lundberg *et al*., 2011; Totzeck *et al*., 2012; Bailey *et al*., 2014). However, *in vitro* this conversion process is relatively slow, perhaps explaining why relatively high, supraphysiological nitrite concentrations are required to relax pre-constricted isolated blood vessels (Furchgott and Bhadrakom, 1953; Maher *et al*., 2008; Ormerod *et al*., 2011). We and others have demonstrated that infused nitrite acts as a vasodilator in healthy volunteers (Gladwin *et al*., 2000; Cosby *et al*., 2003; Larsen *et al*., 2006; Dejam *et al*., 2007; Maher *et al*., 2008), with greater potency as an arteriolar vasodilator in forearm resistance vessels of patients with heart failure (HF) compared with those of normal subjects (Maher *et al*., 2013). Although several candidate mechanisms have been postulated, the exact mechanism(s) responsible for bioactivation of nitrite in man remains ill defined.

Under conditions of profound hypoxia and/or acidosis, the conversion of nitrite to NO may occur via acid disproportionation. Under less extreme conditions, various factors have been reported to convert nitrite to NO. These include xanthine oxidoreductase (XOR), aldehyde oxidase, endothelial NOS (eNOS) and haem proteins (Doyle *et al*., 1981; Basu *et al*., 2007; Li *et al*., 2008; Pinder *et al*., 2009; Lundberg *et al*., 2011; Tiso *et al*., 2011; Totzeck *et al*., 2012). Previous studies have demonstrated that mitochondrial aldehyde dehydrogenase 2 (ALDH2) may be an important additional source of nitrite-derived NO in rat heart (Perlman *et al*., 2009) and the vasculature (Golwala *et al*., 2009), but whether the oxidative environment in the vasculature of HF patients may alter the role for ALDH2 remains unknown. Interestingly, ALDH2 is also known to act as a reductase of organic nitrates, such as glyceryl trinitrate (GTN; Chen *et al*., 2002), and currently GTN is used to treat HF patients. However, prolonged exposure of the vasculature to GTN leads to inactivation of ALDH2. Although it has been argued that ALDH2 inactivation is the principal mechanism for the development of nitrate tolerance (Chen *et al*., 2002; DiFabio *et al*., 2003), other investigators have demonstrated hysteresis between the onset/offset of ALDH2 inactivation by GTN and the time course of GTN tolerance (D'Souza *et al*., 2011), potentially permitting the utilization of GTN pre-exposure as an ALDH2-inactivating manoeuvre without induction of GTN tolerance.

Herein, we first explored the putative role of ALDH2 as a contributor to nitrite bioactivation under normoxic and hypoxic conditions, utilizing *in vitro* isolated rat vessels. To translate our findings into the clinical setting, we explored the role of ALDH2 in a proof-of-principle study in HF patients *in vitro* (isolated vessel) and *in vivo* forearm blood flow (FBF; measured by venous occlusion plethysmography). We chose to evaluate HF patients, first because of the potential therapeutic relevance, and second because we have previously shown that *in vivo* arteriolar responses to nitrite are increased in patients with HF versus healthy controls (Maher *et al*., 2013).

## Methods

The animal studies were approved by the UK Home Office and conducted according to the Animals (Scientific Procedures) Act of 1986 and European Commission guidelines. A total of 35 animals were used in the experiments described here. The human investigations conformed to the Declaration of Helsinki and were approved by the South Birmingham Research Ethics Committee (10/H1207/50) and registered with the UK Clinical Research Network (UK CRN 9587). All patients gave written informed consent after satisfying the inclusion and exclusion criteria as described below.

### Tension myography

Male Sprague–Dawley rats (250–300 g) were killed by an overdose of anaesthetic by an i.p. injection of sodium pentobarbital (100 mg·kg^−1^). The thoracic aorta was carefully removed and placed immediately in cold Krebs bicarbonate buffer (pH 7.4, 95% O_2_; 5% CO_2_) of the following composition (mmol·L^−1^): NaCl (119), KCl (4.7), CaCl_2_ (2.5), KH_2_PO_4_ (1.18), MgSO_4_ (1.19), NaHCO_3_ (25.1), glucose (11). Thoracic aortas were cleaned from adhering connective tissue and cut into three to four ring segments. Aortic rings were mounted in a myograph (Multi-Myograph 610M, Danish Myotechnology, Aarhus, Denmark) containing 5 mL Krebs bicarbonate buffer (37°C, pH 7.4) and gassed with 95% O_2_/5% CO_2_. After an equilibration period of 45 min, vessels were normalized as previously described (Madhani *et al*., 2003). Following normalization, each vessel was primed with KCl (4.8 mmol·L^−1^) before a supramaximal concentration of phenylephrine (PE; 10 μmol·L^−1^; Sigma Aldrich, Dorset, UK) was added. Once the contractile tone had stabilized, ACh (1 μmol·L^−1^; Sigma Aldrich) was added to the organ bath to assess the integrity of the endothelium. If the constrictor responses to PE were not maintained or ACh elicited relaxations <50% of the PE-induced pre-contractile tone, the preparation was discarded. Tissues were then washed for 30 min (by addition of fresh Krebs bicarbonate buffer at 15 min intervals) after which cumulative concentrations of PE (0.001–1 μmol·L^−1^) were added to the myograph. These tissues were then washed over 60 min to restore basal tone before being contracted by PE, to approximately 80% of the maximum PE-induced response. Once a stable response to PE was achieved, a cumulative concentration–response curve to sodium nitrite (NaNO_2_; Sigma Aldrich) was constructed (0.001–100 μmol·L^−1^) under normoxic conditions (95% O_2_/5% CO_2_). Under hypoxic conditions (95% N_2_/5% CO_2_; resulting in ≈1% tissue bath O_2_; Maher *et al*., 2008), the vessels were incubated for 30 min before exposure to the EC_80_ of PE (to achieve similar tension observed at 95% O_2_) and the administration of NaNO_2_.

To delineate the role of ALDH2 in nitrite-mediated vasorelaxation, concentration–response curves to NaNO_2_ were constructed before and after 30 min of incubation with cyanamide (1 mmol·L^−1^; ALDH2 inhibitor; DiFabio *et al*., 2003; Sigma Aldrich) or propionaldehyde (1 mmol·L^−1^; ALDH2 substrate that acts as a competitive inhibitor; DiFabio *et al*., 2003; Sigma Aldrich) under normoxic and hypoxic conditions.

To determine whether GTN pre-exposure of tissues leads to an attenuation of the response to nitrite (via inactivation of ALDH2), aortic rings were exposed to hypoxic conditions as described earlier and then incubated in the presence or absence of 30 or 100 μmol·L^−1^ of GTN for a period of 1 h (Keith *et al*., 1982). Thereafter, blood vessels were washed every 15 min for 1 h. After pre-contraction to the EC_80_ of PE, a concentration–response curve to NaNO_2_ was constructed.

### *In vitro* and *in vivo* analysis in HF patients

The effect of ALDH2 inhibition on nitrite-mediated vasorelaxation was investigated in HF patients: (i) *in vitro* in isolated resistance vessels obtained from gluteal subcutaneous fat tissue and (ii) *in vivo* by measuring changes in FBF during intra-arterial infusion of sodium nitrite with and without GTN pretreatment (to decrease ALDH2 activity).

### Patient demographics

Patients were grouped as follows: (i) biopsy group (*in vitro* myography; *n* = 16); (ii) plethysmography study: saline group (*n* = 8) and GTN group (*n* = 13); Table 1). *Inclusion criteria*: systolic HF [left ventricular ejection fraction (LVEF) <40], aged between 40 and 80 years, and non-smoker. *Exclusion criteria*: treatment with long-acting nitrates, past history of adverse reactions to organic nitrates, hypotension (systolic BP <110 mmHg), concomitant warfarin/clopidogrel therapy or with bleeding diathesis, and obstructive sleep apnoea. All subjects were asked to refrain from alcohol, and foods with a high nitrate/nitrite content for 24 h, and caffeine for 12 h before the study.

**Table 1:** Patient demographics

	*In vitro* analysis	*In vivo* analysis	
	Biopsy-only group (*n* = 16)	Saline group (*n* = 8)	GTN group (*n* = 13)
Age (years)	64.5 ± 3.8	62 ± 4.0	66 ± 3.3
Male gender, *n* (%)	13 (81)	7 (88)	12 (92)
Mean weight (kg)	78.3 ± 3.8	74.6 ± 2.5	82.3 ± 3.3
Body mass index (kg·m^–2^)	27.8 ± 1.5	25.4 ± 0.5	27.1 ± 0.9
Ejection fraction (%)	26.4 ± 2.3	25.1 ± 2.5	27 ± 2.1
NYHA class			
I	2	1	1
II	6	4	10
III	8	3	2
Heart rate (beats·min^-1^)	72 ± 2.8	62 ± 4.4	62 ± 2.0
MABP (mmHg)	95 ± 4.1	89 ± 2.9	88 ± 2.2
Aetiology, *n* (%)			
Dilated cardiomyopathy	8 (50)	5 (62)	6 (46)
Ischaemic cardiomyopathy	6 (38)	3 (38)	6 (46)
Other	2 (13)	0	1 (8)
Medication, *n* (%)			
ACEI/AT_2_ receptor antagonists	15 (94)	8 (1 00)	12 (92)
β-Blockers	10 (63)	5 (62)	10 (77)
Spironolactone/eplerenone	10 (63)	3 (38)	3 (23)
Loop diuretic	12 (75)	4 (50)	8 (62)
Aspirin	12 (75)	4 (50)	10 (77)

Data expressed as mean ± SEM.

ACEI, ACE inhibitors; MABP, mean arterial BP; NYHA, New York Heart Association classification.

### Effect of ALDH2 inhibition in isolated resistance vessels

In a subgroup of nitrite/nitrate naïve HF patients (i.e. no infusions or treatment of NaNO_2_ and/or GTN), subcutaneous gluteal fat biopsies were obtained under local anaesthetic (2% lidocaine) and placed in cold Krebs bicarbonate buffer as previously described (Greenstein *et al*., 2009). Immediately after harvesting the subcutaneous fat biopsies, small resistance arteries (∼250 μm ID; 2 mm long) were isolated from the fat, and mounted onto a myograph (Greenstein *et al*., 2009). In most cases, two vessels from each biopsy were studied. The myograph protocol for normalization, priming of vessels with KCl, the assessment of supramaximal concentration of PE and the endothelial integrity, was assessed as above. Cumulative concentration–response curves to NaNO_2_ were constructed (0.001–100 μmol·L^−1^) under normoxic and hypoxic conditions as described earlier.

To investigate whether attenuation of nitrite efficacy during hypoxia was caused by the effects of ALDH2 inhibition on downstream signalling in the NO cascade, concentration–response curves to the NO donor, spermine-NONOate [N-(2-aminoethyl)-N-(2-hydroxy-2-nitrosohydrazino)-1,2-ethylenediamine (Sper/NO); 0.001–1 μmol·L^−1^; Calbiochem (EMD Millipore)], were constructed in the absence and presence of the ALDH2 inhibitor, cyanamide, under hypoxic conditions.

### The effect of ALDH2 inactivation by GTN on FBF

To determine the effects of ALDH2 inactivation by GTN on arteriolar responsiveness in patients from HF, forearm vascular responsiveness was assessed using the plethysmography. The studies on HF patients were performed in a quiet temperature-controlled (22–24°C) laboratory. All subjects were placed in a semi-recumbent position enabling the administration of hypoxia to participants. Changes in FBF were determined in both arms by venous occlusion plethysmography (DE Hokanson, Bellevue, WA, USA) and BP, heart rate (HR) and oxygen saturation were monitored as previously described (Maher *et al*., 2008). A 27 gauge arterial needle (Coopers Engineering, Birmingham, UK) mounted onto a 16 gauge epidural catheter and sealed with dental wax was then inserted aseptically into the brachial artery of the non-dominant arm and was kept patent by the continuous infusion of normal saline (Baxter Healthcare, Deerfield, IL, USA; 0.9%). An i.v. cannula (20 gauge) was inserted in the contralateral arm for venous blood sampling. Saline was infused via the intra-arterial line at 1 mL·min^-1^ for 10 min for baseline measurements before infusion of NaNO_2_. Data were acquired from both arms, and any changes observed were corrected for those occurring in the contralateral control arm and presented as a ratio of FBF (FBF-R) during infusion compared with baseline (Maher *et al*., 2008).

**Figure 1:**
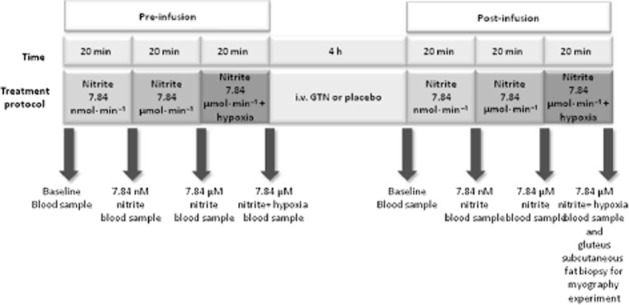
Schematic protocol of the plethysmography study.

As depicted in Figure 1, following 10 min of rest, two doses of NaNO_2_ (Martindale Pharmaceuticals, Buckingham, UK) were infused into the brachial artery of the non-dominant arm (784 nmol·min^−1^ then 7.84 μmol·min^−1^ for 20 min each; the infusion rate was 1 mL·min^−1^ as described previously; Maher *et al*., 2008). FBF was measured in both arms. Following the second dose of NaNO_2_ (7.84 μmol·min^−1^), the patients switched from breathing room air to inspiring 12% oxygen via a facemask connected to a two-way valve. Upon achieving target oxygen saturation of 83–88%, the study proceeded with the 7.84 μmol·min^−1^ infusion of nitrite for a further 10 min. Thereafter, patients were randomized to receive either an i.v. infusion of 10 μg·min GTN (to decrease ALDH2 activity; Merck-Lipha Pharmaceuticals Ltd, Middlesex, UK) as described previously (Philpott *et al*., 2007) or saline (the placebo group; active ALDH2) for 4 h (15 mL·h^−1^). Following a 30 min washout period (saline at 1 mL·min^−1^), repeat infusions of NaNO_2_ were administered during normoxia and hypoxia as described earlier.

Upon completion of the plethysmography protocol, the intra-arterial needle was removed. Participants were allowed to rest for 10 min following which a subcutaneous fat biopsy was obtained (Figure 1) as described earlier. Philpott *et al*. (2007) have previously reported that low-dose GTN infusion rapidly inactivates ALDH2 and that this inactivation occurs before the development of significant nitrate tolerance. Therefore, to determine whether the infusion of GTN had induced significant nitrate tolerance, GTN vasodilator concentration–response curves were compared in resistance vessels taken via gluteal biopsy in patients from the saline (placebo group; active ALDH2) and GTN (inactive ALDH2) infusion group. Briefly, the isolated tissue resistance vessels were mounted in a myograph, the vascular rings were assessed for endothelial integrity and then contracted sub-maximally with PE and cumulative concentration–response curves were constructed for GTN (0.001–1 μmol·L^−1^) from the saline and GTN infusion HF patient group respectively.

### Blood samples

Venous blood samples were taken at eight time points during the plethysmography study (Figure 4) for determination of venous methaemoglobin (MetHb) and pH (blood gas analyser Bayer Rapidlab 865, Siemens, NY, USA), and determination of total plasma 8-iso prostaglandin F_2α_ (8-isoprostane EIA assay kit protocol, Cayman Chemical, Ann Arbor, MI, USA).

### Isolation of mitochondrial fraction

Rat aortic vessels that were treated as described earlier in the tension myography studies were immediately snap frozen at the end of the protocol for isolation of the mitochondrial fraction. Frozen thoracic aorta was suspended in the mitochondrial buffer containing 10 mmol·L^−1^ MOPS (pH 7.2), 10 mmol·L^−1^ KCl, 1.5 mM MgCl_2_, 1 mmol·L^−1^ EDTA, 10 μg·mL^−1^ leupeptin, 10 μg·mL^−1^ aprotinin and 0.25 mol·L^−1^ sucrose, and gently homogenized with a Dounce homogenizer (30 strokes) as previously described (Paneni *et al*., 2013). The homogenate was centrifuged at 750× *g* for 10 min at 4°C to remove nuclei and unbroken cells, and the supernatant was subsequently centrifuged at 10 000× *g* for 15 min. The resultant mitochondrial pellet was used for the ALDH2 assay kit (see ALDH2 activity assay for details).

### Mitochondrial ALDH2 activity assay

ALDH2 activity was determined in mitochondria isolated from rat thoracic aorta following solubilization and extraction as specified in the manufacturer's recommendations (mitochondrial ALDH2 activity assay kit; Abcam, Cambridge, UK). The homogenate was then incubated on ice for 20 min and centrifuged at 16 000× *g* for 20 min at 4°C. Protein concentration of the supernatant was determined and 20 μg of protein was used to detect ALDH2 activity. In this assay, the generation of NADH is coupled to the 1:1 reduction of a reporter dye to yield reaction product concentration, which was monitored by measuring the absorbance increase at 450 nm.

### Statistical analysis

All data are expressed as mean ± SEM, and significance was accepted with *P* < 0.05. For the myography analysis, concentration–response curves were analysed using two-way anova. For the *in vivo* FBF analysis, one-way anova repeated measures coupled with a Bonferroni *post hoc* test was used to compare the effects of pre-saline/GTN or post-saline/GTN infusion treatment. A paired non-parametric test (Wilcoxon signed-rank test) was used to compare FBF following hypoxia between pre-GTN infusion and post-GTN infusion. Statistical analysis was undertaken using Prism software (version 4.0, GraphPad Software, La Jolla, CA, USA).

## Results

### Evaluation of the role of ALDH2 in nitrite-mediated bioactivation in rat aorta

**Figure 2:**
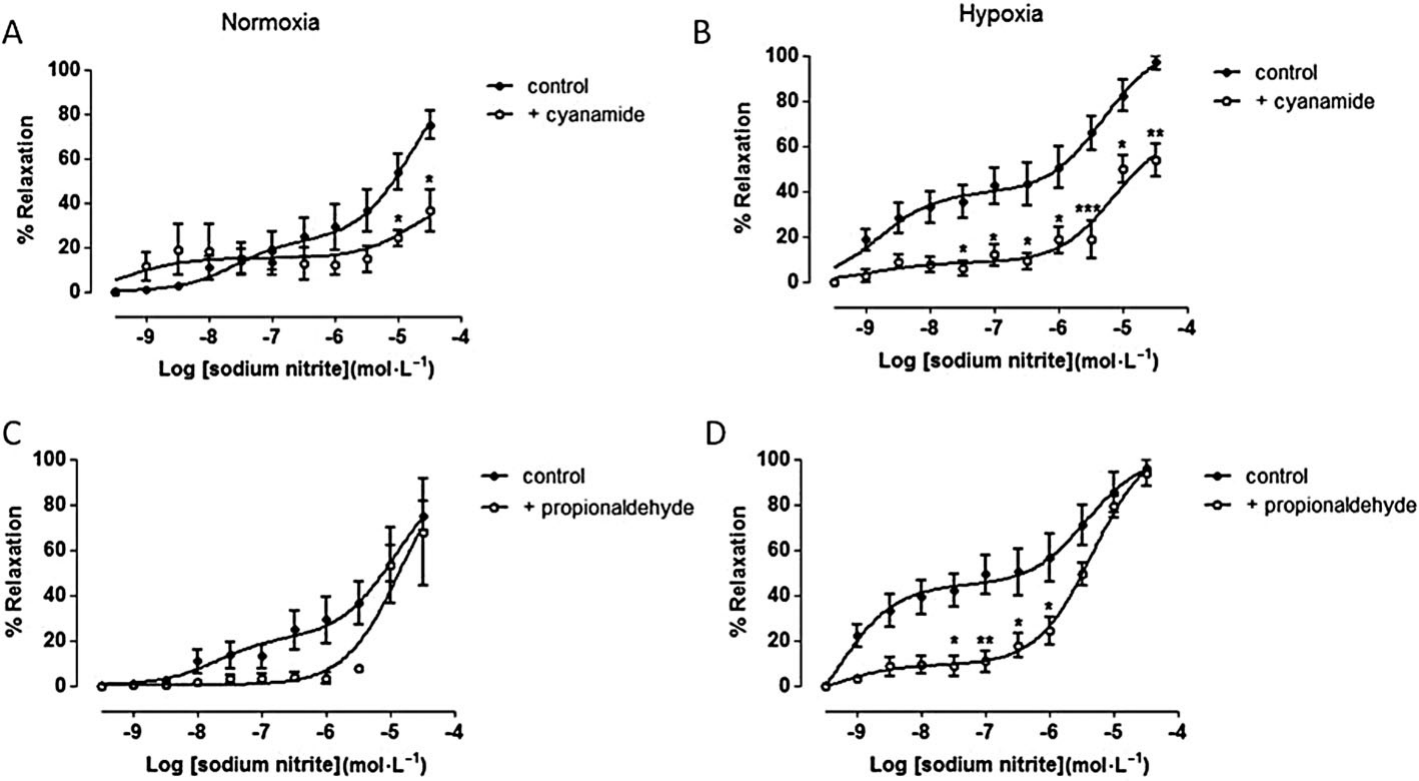
ALDH2 inhibition attenuates nitrite-induced vasorelaxation in rat aortae. Concentration–response curves to sodium nitrite in the presence or absence of ALDH2 inhibitor, cyanamide, during (A) normoxia (*n* ≥ 5), (B) hypoxia (*n* ≥ 5), and ALDH2 substrate, propionaldehyde, during (C) normoxia (*n* ≥ 5) and (D) hypoxia (*n* ≥ 5). Relaxation is expressed as mean ± SEM percentage reversal of PE-induced tone. *P < 0.05, **P < 0.01 and ***P < 0.001 control versus cyanamide or propionaldehyde by two-way anova.

As depicted in Figure 2, the vasorelaxant response to NaNO_2_ displays a biphasic concentration–response relationship. As shown in Figure 2A, following incubation of vessels with the ALDH2 inhibitor cyanamide during normoxic conditions, vasorelaxation by NaNO_2_ was paradoxically enhanced at nanomolar concentrations and significantly inhibited at higher NaNO_2_ concentrations (10 and 30 μM) when compared with controls (*P* < 0.05; by two-way anova). Hypoxia enhanced nitrite-induced relaxation but pretreatment with cyanamide during hypoxia caused a significant attenuation of nitrite's efficiency to relax blood vessels at all concentrations tested when compared with the control (*P* < 0.05; by two-way anova; Figure 2B). Similar results were obtained using the ALDH2 substrate, propionaldehyde. Incubation of vessels with propionaldehyde under normoxic conditions blunted the vasorelaxant effect of low concentrations of nitrite without affecting its efficacy at higher concentrations, but these differences did not reach statistical significance (*P* < 0.05; by two-way anova; Figure 2C). However, under hypoxic conditions, propionaldehyde significantly attenuated the response to nitrite in the nano- to micromolar concentrations range when compared with control (*P* < 0.05; by two-way anova; Figure 2D) without affecting maximal relaxation induced by the highest concentration of nitrite.

### Effect of ALDH2 inhibition on nitrite-mediated vasorelaxation in resistance vessels from HF patients

**Figure 3:**
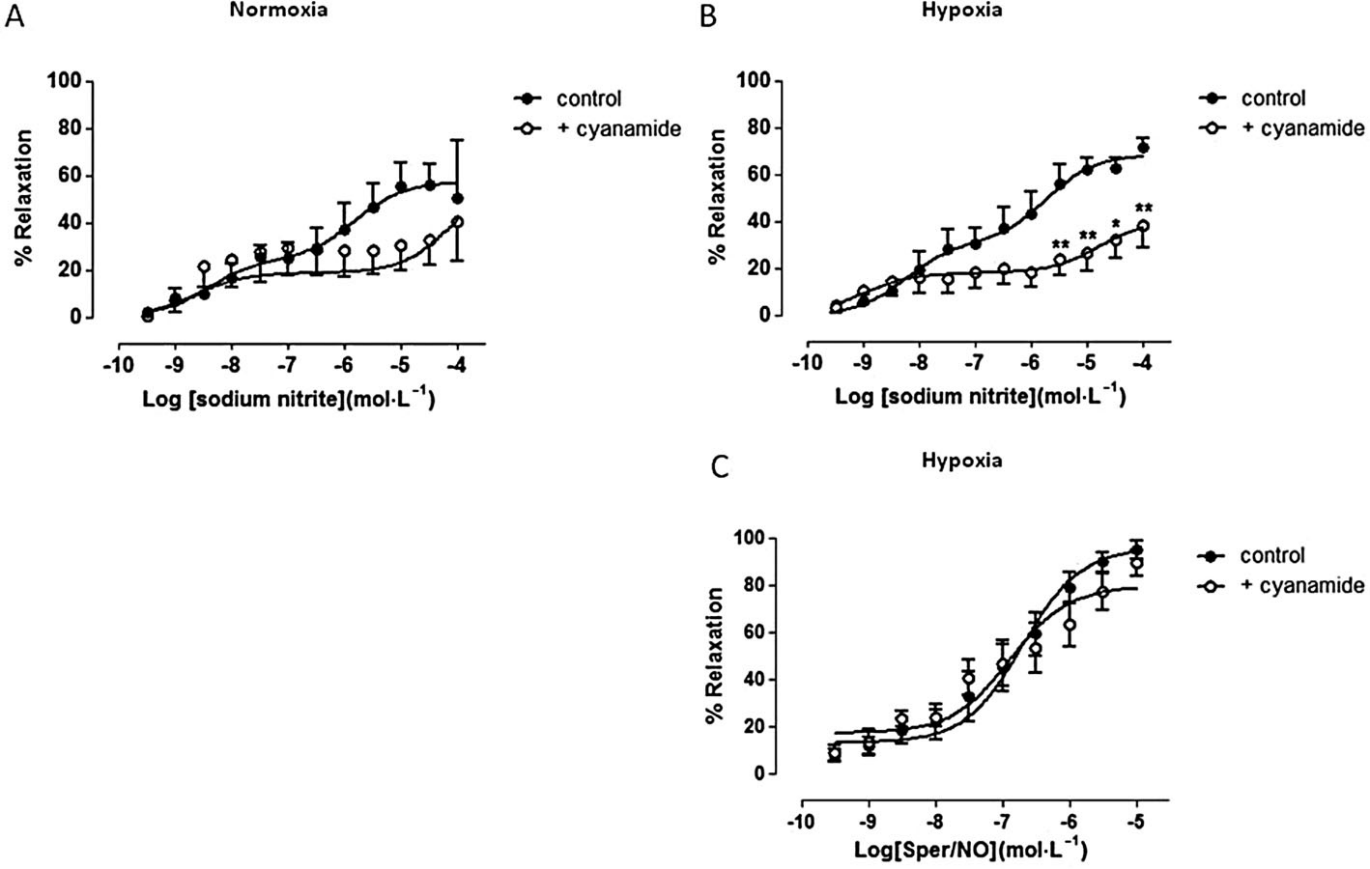
ALDH2 inhibition decreases nitrite-induced vasorelaxation in resistance vessels from HF patients. Concentration–response curve to sodium nitrite in the presence or absence of cyanamide during (A) normoxia (*n* = 7) and (B) hypoxia (*n* = 9). (C) Concentration–response curve to Sper/NO in the presence or absence of cyanamide during hypoxic conditions (*n* ≥ 6). Relaxation is expressed as mean ± SEM percentage reversal of PE-induced tone. *P < 0.05 and **P < 0.01 control versus cyanamide by two-way anova.

Under both normoxic and hypoxic conditions, NaNO_2_ caused concentration-dependent relaxation of resistance vessels from HF patients (Figures 3A and B). During normoxic conditions, pretreatment with cyanamide tended to shift the NaNO_2_ concentration–response curve to the right but this was not statistically significant (Figure 3A). Under hypoxic conditions, pretreatment with cyanamide caused a marked and concentration-dependent attenuation of the relaxation responses to NaNO_2_ when compared with control (*P* < 0.05; by two-way anova; Figure 3B). To assess whether this effect was nitrite specific, concentration–response curves to the NO donor Sper/NO were constructed in the presence or absence of cyanamide. Cyanamide had no inhibitory activity on the vasorelaxant responses to Sper/NO under hypoxic conditions (Figure 3C).

**Figure 4:**
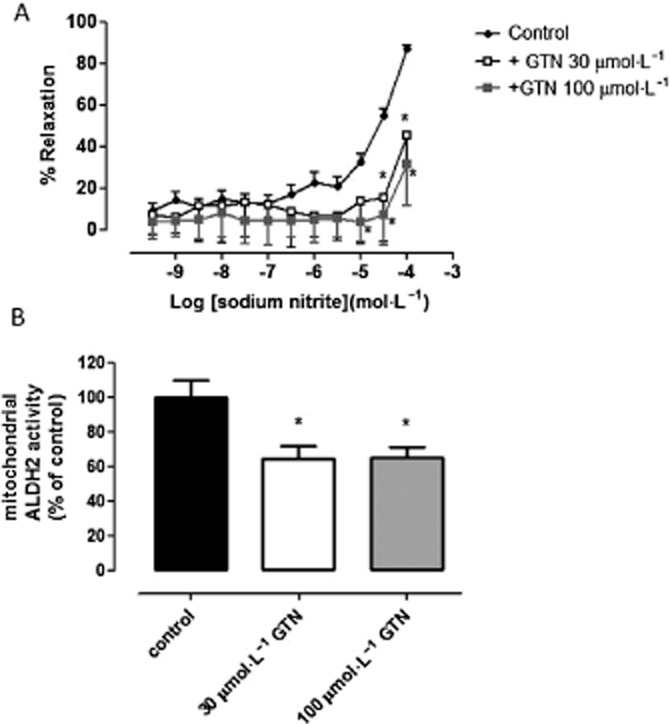
Tolerance-independent inactivation of ALDH2 attenuates vasorelaxation in rat aortae. (A) Concentration–response curve to NaNO_2_ in the presence or absence of GTN during hypoxic conditions. Relaxation is expressed as mean ± SEM percentage reversal of PE-induced tone (*n* = 10); *P < 0.05, ***P < 0.001 versus control, two-way anova. (B) The effect of sodium nitrite (control), in the presence or absence of GTN during hypoxic conditions, on mitochondrial ALDH2 activity (mean ± SEM from *n* = 4–6 animals; *P < 0.05 vs. control by one-way anova).

### Effect of GTN pretreatment on nitrite-mediated vasorelaxation in rat aorta

In addition to the aforementioned inhibitor/substrate experiments, we also assessed the effects of GTN (used as a tool to decrease ALDH2 activity) on nitrite-mediated vasorelaxation. Using a well-established *in vitro* model of nitrate tolerance in rat isolated aorta (Keith *et al*., 1982; Irvine *et al*., 2007), blood vessels were pretreated with GTN (30 or 100 μmol·L^−1^) for 1 h followed by construction of a concentration–response curve to NaNO_2_ under hypoxic conditions. Pretreatment with GTN (30 and 100 μmol·L^−1^) significantly attenuated the response to NaNO_2_ compared with controls (*P* < 0.05; by two-way anova; Figure 4A), particularly at higher concentrations. As shown in Figure 4B, mitochondrial ALDH2 activity was significantly decreased following pretreatment with GTN (30 and 100 μmol·L^−1^) when compared with control (NaNO_2_; *P* < 0.05; by one-way anova).

### Effects of GTN-induced ALDH2 inactivation on nitrite-mediated vasodilatation in the human forearm

**Figure 5:**
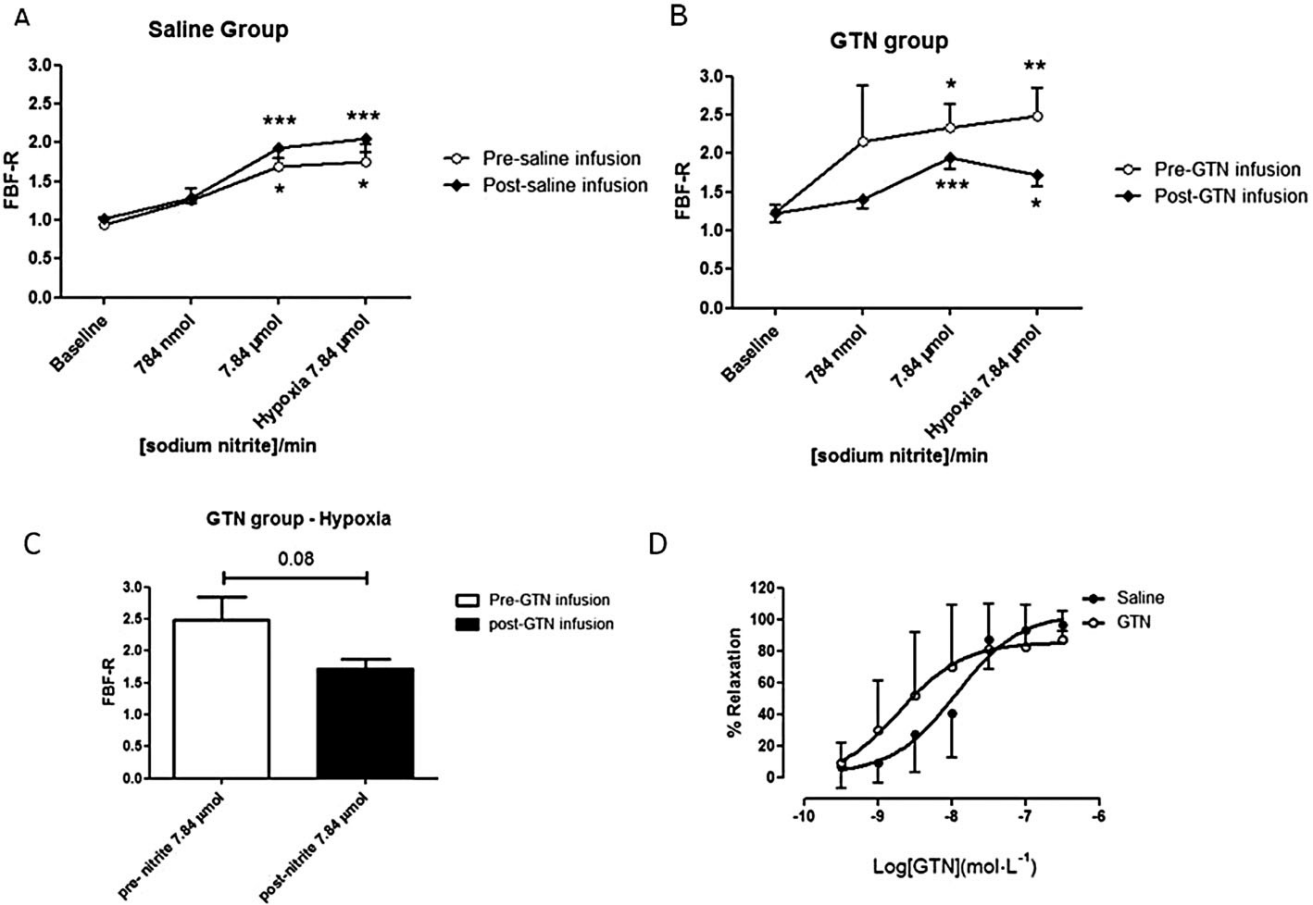
GTN infusion attenuates nitrite-induced vasorelaxation in the resistance vasculature of HF patients. Forearm vasodilator measurements following nitrite infusion in HF patients subjected to 4 h infusion of (A) saline (*n* = 8 patients) or (B) GTN (*n* = 11 patients) treatment. *P < 0.05, **P < 0.01, ***P < 0.001 compared with baseline. (C) Comparison of pre- and post-GTN infusion following 7.84 μmol·min^−1^ sodium nitrite during hypoxic conditions (*n* = 8 and *n* = 11 patients, respectively; *P* = 0.08). (D) Concentration–response curve to GTN in isolated resistance vessels from HF patients (saline *n* = 7; GTN *n* = 11 patients).

FBF corrected for changes in the non-infused arm (FBF ratio) increased dose-dependently with NaNO_2_ in the saline (placebo) and GTN groups respectively (Figure 5A and B). As depicted in Figure 5A, one-way anova repeated measures showed that 7.84 μmol·min^−1^ nitrite significantly increased FBF in the pre-saline infusion group from 0.94 ± 0.09 at baseline to 1.69 ± 0.21 during normoxic (*P* < 0.05 compared with baseline) and 1.76 ± 0.22 during hypoxic conditions (*P* < 0.05 compared with baseline; Figure 5A). Following 4 h i.v. saline infusion (placebo group; active ALDH2), a similar profile in FBF-R response to NaNO_2_ was observed when compared with pre-saline infusion. Nitrite 7.84 μmol·min^−1^ significantly increased FBF in the post-saline infusion group from 1.02 ± 0.04 at baseline to 1.93 ± 0.14 during normoxic (*P* < 0.001 compared with baseline) and 2.05 ± 0.18 during hypoxic conditions (*P* < 0.001 compared with baseline).

In the GTN group (Figure 5B), prior to administration of i.v. GTN, one-way anova repeated measures showed that 7.84 μmol·min^−1^ nitrite infusion significantly increased FBF from 1.24 ± 0.08 at baseline to 2.34 ± 0.29 during normoxic (*P* < 0.05 compared with baseline) and 2.48 ± 0.36 (*P* < 0.01 compared with baseline) during hypoxic conditions. Following 4 h infusion of GTN to inactivate ALDH2, although 7.84 μmol·min^−1^ nitrite infusion significantly increased FBF from 1.20 ± 0.11 (baseline) to 1.95 ± 0.15 during normoxic conditions (*P* < 0.001 when compared with baseline) and 1.72 ± 0.15 during hypoxia (*P* < 0.05 when compared with baseline). However, GTN treatment tended to attenuate the increase in FBF following nitrite infusion (7.84 μmol·min^–1^) when compared with the pre-GTN infusion. Using a paired non-parametric test (Wilcoxon signed-rank test), we compared pre- and post-GTN infusion for each of baseline and 784 nmol and 7.84 μmol at normoxic conditions and at hypoxic conditions respectively. There was no significant difference between pre-GTN versus post-GTN infusion at baseline (1.24 ± 0.09 and 1.20 ± 0.11, respectively; *P* = 0.79), pre-GTN versus post-GTN infusion with 784 nmol·min^−1^ (2.16 ± 0.72 and 1.35 ± 0.10, respectively; *P* = 0.34), or at pre-GTN versus post-GTN infusion with 7.84 μmol·min^−1^ (2.34 ± 0.29 and 1.95 ± 0.15, respectively; *P* = 0.28). During hypoxic conditions, GTN treatment attenuated the increase in FBF following nitrite infusion (7.84 μmol·min^−1^) but this effect did not reach statistical significance (2.48 ± 0.36 and 1.72 ± 0.15, pre- and post-GTN infusion, respectively; *P* = 0.08; Figure 5C).

### GTN infusion does not induce tolerance in resistance vessels

To confirm that our low-dose GTN infusion protocol did not induce systemic nitrate tolerance, a gluteal biopsy was obtained at the end of the plethysmography protocol and isolated resistance vessels were used for myography experiments. There was no attenuation of the vasodilator response to GTN compared with that in the control vessels isolated from the saline infusion group (*P* = 0.87; Figure 5D).

### Assessment of haemodynamics, venous pH, 8-isoprostanes and methaemoglobin formation during nitrite infusion in HF patients

HR and mean arterial BP (MABP) did not alter significantly from baseline following administration of NaNO_2_ in the saline and GTN groups (Table 2). Arterial oxygen saturations remained stable throughout the normoxia period in the saline group, but fell significantly following administration of 12% oxygen in both the saline and GTN groups (from 97 ± 0.5% to 87 ± 1.1%, *P* < 0.001 and from 97 ± 0.3 to 87 ± 0.6, *P* < 0.001 respectively). There was no significant change in either venous pH (saline group *n* = 5–7; GTN group *n* = 9–11; *P* < 0.05) or 8-isoprostane levels (saline group *n* = 4–6; GTN group *n* = 7–9; *P* < 0.05) in both the saline and GTN groups.

**Table 2:** Assessment of haemodynamic, venous pH, 8-isoprostane and methaemoglobin

	Saline group				GTN group			
	BL	784 nmol·min^−1^	7.84 μmol·min^−1^	Hypoxia + 7.84 μmol·min^−1^	BL	784 nmol·min^−1^	7.84 μmol·min^−1^	Hypoxia + 7.84 μmol·min^−1^
HR (bpm)	62 ± 4.1	64 ± 4.1	65 ± 4.8	68 ± 5.7	62 ± 2.3	62 ± 2.2	60 ± 1.6	6.4 ± 0.9
MABP (mmHg)	84 ± 1.9	85 ± 2.9	78 ± 2.5	80 ± 2.6	83 ± 2.8	83 ± 2.6	86 ± 2.5	85 ± 2.6
Arterial O_2_ Sat (%)	97 ± 0.5	97 ± 0.5	97 ± 0.5	87 ± 1.1*	97 ± 0.3	98 ± 0.3	98 ± 0.3	87 ± 0.6*
pH	7.37 ± 0.02	7.39 ± 0.01	7.40 ± 0.01	7.41 ± 0.01	7.37 ± 0.01	7.38 ± 0.01	7.38 ± 0.01	7.39 ± 0.01
Isoprostanes (ng·L^−1^)	5.96 ± 0.55	7.42 ± 0.93	7.13 ± 0.39	7.29 ± 0.88	7.61 ± 1.00	9.14 ± 1.75	7.54 ± 0.83	9.45 ± 2.45
MetHb (% of total Hb)	0.31 ± 0.04	0.61 ± 0.08	1.39 ± 0.21*	1.63 ± 0.17*	0.37 ± 0.06	0.58 ± 0.07	1.51 ± 0.18*	1.57 ± 0.15*

Data expressed as mean ± SEM.

* *P* < 0.0001.

BL, baseline; GTN, glyceryl trinitrate; HR, heart rate; MABP, mean arterial blood pressure; MetHb%, methaemoglobin measurements following sodium nitrite infusion in the saline or GTN group; O_2_, arterial oxygen saturation; pH, plasma 8-isoprostane.

Following 4 h saline infusion, venous MetHb levels increased during escalating NaNO_2_ dose infusions from 0.31 ± 0.04% to 1.39 ± 0.21% (baseline and 7.84 μmol·min^−1^ nitrite, normoxia) of total Hb. Highest levels were reached following 7.84 μmol·min^−1^ nitrite infusion during hypoxic conditions (1.63 ± 0.17%; *P* < 0.001; *n* = 5–7). In the GTN group, a similar trend was observed, MetHb levels increased from 0.37 ± 0.06% to 1.51 ± 0.18% (baseline and 7.84 μmol·min^−1^ nitrite, respectively, normoxia) with the highest levels of MetHb (1.57 ± 0.15%; *P* < 0.001; *n* = 9–11) reached following 7.84 μmol·min^−1^ nitrite infusion during hypoxic conditions. There was no significant difference between the two groups. Pre-infusion data were also similar for the haemodynamics and MetHb pre-saline/GTN (data not shown).

## Discussion

We previously demonstrated that intra-brachial artery infusion of nitrite causes forearm vasodilatation in both healthy subjects and in HF patients, with greater potency in the latter (Maher *et al*., 2013), but the mechanism(s) underlying these effects remains incompletely understood. Based on these observations, we here explored the role of ALDH2 in nitrite bioactivation under normoxia and hypoxia in both rats and human vasculature. Our findings suggest that ALDH2 plays a major role in nitrite-mediated vasorelaxation *in vitro*. However, this effect was clearly less marked *in vivo*, and we postulate that this could be due to the presence of multiple *in vivo* mechanisms.

Several nitrite reductases have been identified, including eNOS, XOR, aldehyde oxidase and haem proteins such as deoxyhaemoglobin and myoglobin, to bioactivate nitrite under hypoxia (Doyle *et al*., 1981; Basu *et al*., 2007; Pinder *et al*., 2009; Lundberg *et al*., 2011; Tiso *et al*., 2011; Baliga *et al*., 2012; Ghosh *et al*., 2013). All of these agents alone and/or in combination are capable of bioconverting nitrite to NO *in vitro*, but their physiological role remains incompletely understood. Previous studies have reported that ALDH2 may be an important source of nitrite-derived NO in the heart (Perlman *et al*., 2009) and vasculature (Badejo *et al*., 2010), but whether the oxidative environment in the vasculature alters the effects of ALDH2 on nitrite-mediated vasorelaxation remains unclear. Therefore, in the present study we first explored the role of ALDH2 in nitrite-mediated vasorelaxation *in vitro* in rat isolated conduit vessels (aorta) during normoxic and hypoxic conditions. Our *in vitro* data showed that the ALDH2 inhibitor, cyanamide and the ALDH2 substrate, propionaldehyde significantly reduced the potency of nitrite under hypoxic conditions. These data indicate the potential for ALDH2 to be involved in hypoxic nitrite vasodilator responses.

Much of our current understanding about nitrite's mode of action as a vasodilator is based on animal experimental work and considerably less information is available on the mechanism of vasodilatation by nitrite in human tissue. Nevertheless, studies in healthy human subjects have shown that nitrite causes marked venodilatation and moderate dose-dependent arteriolar dilatation, and these effects are augmented by hypoxia or exercise in healthy subjects (Cosby *et al*., 2003; Maher *et al*., 2008). We have previously shown that *in vivo* arteriolar responses to nitrite are increased in patients with HF versus healthy controls (Maher *et al*., 2013). Because of the potential therapeutic relevance of these findings, we now chose to translate these observations to the clinical setting by evaluating the role of ALDH2 in HF patients *in vitro* and *in vivo*. Because the vascular responses to intra-arterial nitrite are most accurately assessed by measuring changes in FBF (i.e. resistance vessel effects), we first investigated whether the effects of ALDH2 inhibition on nitrite-mediated vasorelaxation observed in the rat isolated conduit vessels were replicated *in vitro* in resistance vessels from patients with HF. We confirmed that the inhibition of ALDH2 significantly attenuates nitrite-mediated vasorelaxation during hypoxic conditions. Importantly, cyanamide did not alter the potency of the NO donor Sper/NO, ruling out non-specific effects of this inhibitor on NO bioactivity. These observations concur with functional data obtained by Huellner *et al*. (2008), where pharmacological inhibition of ALDH2 did not alter the potency to the NO donor DEA/NO in isolated human veins. To our knowledge, this is the first study in man to assess the effects of NaNO_2_ in isolated resistance vessels from HF patients. Interestingly, the magnitude difference in vasorelaxation response to NaNO_2_ observed in the normoxic versus hypoxic group of blood vessels from HF patients was similar. Moreover, the response to NaNO_2_ when comparing rat thoracic aorta versus HF patient resistance vessels was also different. This might be related to different vascular beds, species differences or the heterogeneity in vascular response between healthy and pathophysiology tissues. Regarding the latter, based on our previous findings (Maher *et al*., 2013), we believe that the reason why the magnitude difference in nitrite-induced relaxation is not that different between normoxia versus hypoxia in isolated resistance vessels from HF patients when compared with conduit vessels (thoracic aorta) from healthy rats is because, first, the vasomotor response to nitrite is altered and enhanced and therefore the magnitude is not that different when compared with hypoxia as suggested from our previous *in vivo* studies (Maher *et al*., 2013).

Previous studies have reported that prior GTN exposure induces nitrate tolerance in association with attenuated ALDH2 activity in the vasculature (DiFabio *et al*., 2003; Huellner *et al*., 2008; D'Souza *et al*., 2011). Therefore, using a well-established *in vitro* model of nitrate tolerance in rat isolated aorta (Keith *et al*., 1982; Irvine *et al*., 2007), we demonstrated attenuation of nitrite-mediated vasorelaxation following pretreatment with GTN during hypoxic conditions. To substantiate the role of ALDH2 as a key effector in nitrite-mediated vasorelaxation, we here show that this attenuation in vascular response is associated with reduced mitochondrial ALDH2 activity.

It is important to recognize that there are 19 known human ALDH isoenzymes, of which only a few, such as ALDH2 (Koppaka *et al*., 2012), have been thoroughly characterized biochemically and by susceptibility to pharmacological inhibition. To date, no antagonists have been developed to specifically inhibit each ALDH isoenzyme without affecting others. Moreover, clear sensitivity differences to different ALDH2 inhibitors have been reported, for example, GTN bioactivation in rat liver was sensitive to chloral hydrate but not to daidzin (Kollau *et al*., 2005). In the current study, we chose to use cyanamide (an irreversible inhibitor) and propionaldehyde (a reversible competitive inhibitor) as both are capable of inhibiting ALDH and have been used to examine the role of ALDH2 in organic nitrate-induced vasorelaxation in rat aorta (Chen *et al*., 2002; DiFabio *et al*., 2003). Notwithstanding possible species differences in sensitivity to both inhibitors and vasodilators (including further differences in bioactivation mechanisms), we further complemented these results by a mechanism-based ALDH2 inhibition approach employing an *in vitro* tolerance model and have reached the same conclusion, that is, that ALDH2 inhibition exerts inhibitory actions of NaNO_2_-mediated vasorelaxation *in vitro*.

Assessing the involvement of specific enzymes in mediating the vasodilator effects of nitrite in man *in vivo* remains a challenge. The ALDH2 inhibitor disulfiram is commonly used in clinical studies without ill effect (Johansson, 1992; Mackenzie *et al*., 2005), but its use in HF patients is contra-indicated (Huffman and Stern, 2003). Therefore, in the present study we elected to use a low-dose GTN infusion as it has been previously reported to rapidly inactivate vascular ALDH2, and that this inactivation occurs prior to the development of significant nitrate tolerance and prior to detectable impairment of GTN bioconversion (Philpott *et al*., 2007). We measured the vasodilator activity of nitrite in HF patients and tested whether pre-infusion of GTN (in order to decrease ALDH2 activity) attenuates forearm vasodilator responses to nitrite during normoxic and hypoxic conditions. Despite the well-recognized presence of both endothelial dysfunction and NO resistance in HF patients (Marti *et al*., 2012), adequate vascular activity in response to nitrite was observed in our study. During normoxic conditions, we found an approximate twofold increase in forearm vasodilator responses following 7.84 μmol·min^−1^ of NaNO_2_ in both the saline and GTN group compared with their baseline responses. These results are similar to our previous findings in healthy volunteers (Maher *et al*., 2008). Under hypoxic conditions, inhibition of ALDH2 with GTN (post-GTN infusion group) tended to reduce the forearm vasodilator response but was not significant when compared with the pre-GTN infusion group. Our observation in gluteal resistance vessels taken at the end of the *in vivo* study confirmed that the low-dose GTN regime had not induced systemic tolerance, which is consistent with a previous study demonstrating that the same regime inhibited ALDH2 without inducing GTN tolerance (Philpott *et al*., 2007).

### Study limitations

Despite strict inclusion/exclusion criteria to standardize experimental procedures, a degree of heterogeneity will exist in the population studied in terms of cause and severity of HF. Furthermore, we did not measure plasma levels of nitrite/nitrate as the results are likely to have been affected by the local and systemic metabolism of GTN to nitrite and nitrate, masking any changes to circulating endogenous nitrite/nitrate levels that may have occurred as a result of ALDH2 inhibition. Finally, we were unable to confirm in our *in vivo* studies whether ALDH2 activity was actually attenuated by GTN.

## Conclusions

Our *in vitro* data confirm a role for ALDH2 in nitrite-mediated vasorelaxation during hypoxic conditions in both rat aorta and human resistance vessels. Because nitrite is under consideration as a therapeutic agent, the role for ALDH2 in the bioactivation of nitrite during hypoxic vasodilatation is of importance. Our *in vitro* experiments demonstrate that nitrite could directly affect vasorelaxation without necessitating interaction with blood constituents. However, we cannot discount other non-enzymatic and/or nitrite reductase species (e.g. haem proteins, eNOS, XOR and aldehyde oxidase), which may contribute to the reduction in nitrite during hypoxic conditions *in vitro* (Li *et al*., 2008; Pinder *et al*., 2009; Baliga *et al*., 2012; Totzeck *et al*., 2012; Ghosh *et al*., 2013). Furthermore, although our *in vivo* results demonstrate a (non-significant) trend towards attenuation of the forearm vasodilator response to nitrite following GTN treatment, we suggest that this is probably due to a decrease in ALDH2 activity. However, no firm conclusion can be drawn about the mechanism sub-serving this effect *in vivo*. The fact that this effect was non-significant (*P* = 0.08) may reflect the presence of multiple *in vivo* mechanisms. In particular, we cannot exclude the role for deoxyhemoglobin *in vivo* as blood-borne NO species derived from nitrite may contribute to its vasodilator effects (Angelo *et al*., 2006). In addition, previous work by Gladwin's group has demonstrated that the inhibition of eNOS or XOR does not attenuate nitrite-induced vasodilatation in healthy volunteers (Cosby *et al*., 2003; Dejam *et al*., 2007). However, although it seems that XOR may not contribute to nitrite reduction in health, a recent work by Ghosh *et al*. (2013) reported that XOR does mediate nitrite reduction during pathological conditions (hypertensive animals and patients). Thus, their relative contribution to the hypoxic vasodilator effect of nitrite in man *in vivo*, in particular in patients with HF, remains unresolved and is yet to be fully explored.

Moreover, the observations of the current study suggest that pre-exposure to GTN (even without inducing tolerance) might attenuate nitrite vasodilator effects. This finding may be of clinical importance when considering nitrite as a therapeutic intervention in patients who may have received recent therapy with organic nitrates. Further studies are required to elucidate the extent to which ALDH2 is involved in nitrite reduction to NO *in vivo* in man.

## Abbreviations

ALDH: aldehyde dehydrogenase
eNOS: endothelial NOS
FBF: forearm blood flow
GTN: glyceryl trinitrate
HF: heart failure
MABP: mean arterial BP
MetHb: methaemoglobin
NaNO_2_: sodium nitrite
NO_2_^−^: nitrite
Sper/NO: Spermine-NONOate
XOR: xanthine oxidoreductase
